# Self-efficacy and metacognitive strategies in blended science learning: a self-regulated learning perspective

**DOI:** 10.3389/fpsyg.2026.1780894

**Published:** 2026-04-29

**Authors:** Honglian Xie

**Affiliations:** School of International Studies, Guangdong Open University (Guangdong Polytechnic Institute), Guangzhou, China

**Keywords:** academic achievement, blended learning, metacognitive strategies, self-efficacy, structural equation modeling

## Abstract

With the accelerated adoption of blended learning in higher education science curricula following the global shift toward digitally integrated instruction, science educators face an urgent need to understand how students’ motivational and cognitive self-regulatory processes function across online and face-to-face modalities. Despite this practical imperative, the field lacks empirically validated models specifying how self-efficacy and metacognitive strategies jointly operate to influence science achievement in blended contexts, leaving instructional designers without evidence-based guidance for targeted intervention. This study aimed to examine the mediations and moderations of self-efficacy and metacognitive strategy use in blended science learning contexts for academic achievement. In this study, a total of 449 participants from a university in China completed a validated questionnaire about their self-efficacy in science learning, usage of metacognitive strategies, and academic performance. Structural equation modeling was conducted to analyze the proposed relationship, while mediation analysis employing bias-corrected bootstrapping was also carried out to explore moderation in multiple groups. Results showed that self-efficacy produced a positive direct effect on academic performance (*β* = 0.317, *p* < 0.001) with an indirect effect through metacognitive strategy use (*β* = 0.122, *p* < 0.01) that explained 27.8% of total effect. Multi-group analysis demonstrated that online learning engagement significantly moderated the self-efficacy-metacognitive strategies pathway (Δ*χ*^2^ = 5.38, *p* = 0.020), with stronger associations observed among high-engagement students (*β* = 0.592) compared to low-engagement students (*β* = 0.469). These findings carry direct implications for science educators, demonstrating that fostering self-efficacy beliefs serves as a catalyst not only for direct achievement gains but also for activating metacognitive regulatory processes, and that the level of online engagement functions as a critical boundary condition that amplifies this motivational-strategic linkage. This study contributes an empirically validated dual-pathway model that advances understanding of how motivational and cognitive self-regulation mechanisms operate in technology-enhanced science learning, offering a theoretically grounded basis for designing differentiated instructional interventions in blended science classrooms.

## 1 Introduction

Among the areas that have been influenced by blended learning in science learning is self-regulated learning, which includes self-efficacy as an important element. Self-efficacy generally involves an individual’s feelings of believing in his/her ability to do a particular academic task (Xu et al., 2023a). In science classrooms that embrace learning approaches such as discovery learning, self-efficacy is an important element as it involves students taking responsibility for their learning while at the same time trying to cope with cognitive load that is involved in scientific inquiry. In recent years, self-regulated learning has emerged as a pivotal perspective in understanding science learning achievements because of its robust associations attested in research involving self-regulated learning and science learning achievements (Balazinec et al., 2024).

In addition to motivational beliefs, metacognitive strategies form part of the cognitive architecture whereby students plan, monitor, and conduct self-evaluation of learning activities. Recent studies within technology-enhanced science learning have shown that metacognitive consciousness could be developed in a planned manner through structured interventions, such as the strategic use of artificial intelligence technology to scaffold students’ reflection upon their thinking processes (Ng et al., 2024). In particular, the use of technology within blended learning platforms opens new avenues for developing metacognitive skills, considering that such learning platforms have a natural demand for intentional integration of self-paced online activities with real-time collaborative activities in a synchronous manner (Istenič, 2024). Nonetheless, studies within science learning have provided significant insights within science learning that indicate a great deal of variability in terms of students’ use of metacognitive strategies within blended learning platforms, as preservice teachers as well as students have been seen to have a heterogeneous experience in terms of the cognitive load required in moving between platforms (Yılmaz and Malone, 2020), which is further increased by considerations within institutional infrastructure and learning support systems that present stark disparities within differing learning contexts (Sareen and Mandal, 2024).

Although self-regulated learning in digital learning environments has attracted increasing academic recognition within recent years, several significant research gaps have been identified in current studies in this field. Current meta-analyses have listed a number of strategies and learning technology tools that have a positive effect on self-regulated learning in online and blended learning platforms, but current meta-analyses mainly concentrate on cognitive and metacognitive strategies without considering motivational constructs (Faza and Lestari, 2025). Currently, a satisfactory model of mediations between self-efficacy expectations and metacognitive strategy interactions in learning outcomes is yet to be developed, and such a model needs to specifically take into account scientific learning domains in which knowledge in a particular subject area and epistemological beliefs have a pivotal influence in science learning (Xu et al., 2023b). The distinctiveness of science learning in this regard warrants particular emphasis. Unlike disciplines grounded primarily in textual interpretation and discursive argumentation, where epistemic beliefs center on interpretive authority and perspectival justification, science learning necessitates the coordination of abstract conceptual reasoning with empirical observation, hypothesis testing, and evidence-based inference across both laboratory and digital environments (Schiefer et al., 2022). The epistemic practices inherent in scientific inquiry, such as formulating testable predictions, designing and evaluating experiments, and reconciling theoretical models with observational data, impose metacognitive demands that are qualitatively different from those encountered in humanities or social science disciplines. Indeed, recent scholarship has argued that metacognition in science requires domain-specific theoretical frameworks rather than generic models, precisely because the regulatory processes involved in scientific reasoning are fundamentally tied to empirical epistemic criteria (Willison et al., 2024). In blended science learning contexts, these demands are further compounded by the requirement to transfer regulatory strategies across disparate learning modalities, including self-paced online simulations, synchronous laboratory investigations, and collaborative problem-solving sessions (Sins et al., 2025). Students must continuously calibrate their comprehension monitoring and strategy selection as they transition between hands-on experimental work in face-to-face settings and self-directed conceptual review in online environments. Consequently, metacognitive strategy deployment in science learning cannot be adequately understood through frameworks developed and validated primarily in non-science domains, thereby necessitating discipline-specific investigation of the self-efficacy-metacognition linkage. In spite of meta-analyses within studies that have provided significant evidence for the efficacy of self-regulated learning interventions at all academic tiers, research studies in self-regulated learning have been concentrating merely on interventions in self-regulated learning in terms of overall effect without considering in-depth analysis of particular cognitive components within self-regulated learning interventions in science learning in online platforms.

This study remedies these issues by examining self-efficacy and metacognitive strategy interactions within a blended science learning environment through a self-regulated learning framework. This study theoretically expands upon current understandings of self-efficacy, as it is not only a determinant of academic success but also a motivational component influencing science students’ metacognitive participation in a technology-enhanced learning environment. Through understanding such interactions within a blended learning environment, this research is able to highlight blended science learning in terms of its mechanisms in making self-regulated learning possible in science learning. By identifying the dual-pathway mechanism through which self-efficacy operates in blended science learning and establishing online engagement as a moderating boundary condition, this research provides an empirically grounded basis for science educators to design targeted interventions that simultaneously address motivational and strategic dimensions of self-regulation in science-specific contexts. This research will theoretically assist in gaining a better understanding of self-regulated learning theories while also offering practical suggestions for designing interventions in learning that will assist in developing students’ strategic and motivational components in a science learning context.

## 2 Literature review

From an epistemological viewpoint, self-regulated learning focuses on students’ proactive participation in regulating and governing their cognitive, metacognitive, and motivational engagements in diverse learning contexts. Research studies in science learning have supported the effectiveness of self-regulated learning strategy instruction in improving teachers’ classroom practices as well as students’ learning performance in a sustained manner through teachers as pivotal models of self-regulated strategy use (Arvatz et al., 2025). Within an ever-increasing digital learning context, it is crucial to assume a holistic perspective specifically related to diverse interlocking platforms as a whole, as current students use a myriad of digital platforms in diverse ways that form self-regulated learning acquisition (Domino and Shaffer, 2025). Within science inquiry learning, a salient contrast specifically related to students’ accessibility of metacognitive knowledge versus strategy use has underscored a profound divide specifically related to strategy use, thus hypothesizing that strategy use is a challenge that surpasses merely augmented knowledge acquisition within science inquiry learning activities (Sins et al., 2025). Longitudinal research studies within blended learning institutions have yielded efficacious validation of self-regulated learning strategy use specifically related to diverse learning accomplishments within disciplines (Anthonysamy et al., 2020).

Self-efficacy beliefs have been identified as key building blocks in psychology that influence science learning engagement of students. Various studies carried out in research studies involving learning in undergraduate biology have concluded that self-efficacy has a greater direct effect compared to broad motivational cognitions in academic achievement domains, thus proving its supremacy in determining performance outcomes (der Von Mehden et al., 2025). Innovative learning approaches such as flipped classrooms have been found beneficial in improving science efficacy in preservice teachers, including attitudinal inclusions of improvement in science efficacy in preservice teachers (Ribeirinha and Correia, 2025). Longitudinal studies of science efficacy beliefs across matriculation periods have found that such beliefs are developed through cumulative mastery experiences rather than remaining discrete, showing that perceived science efficacy develops in a non-random fashion across matriculation periods (Hu et al., 2022). Studies of self-efficacy and science learning success in academic milieus have found that such variables are tempered by demographic variables, thus making participation towards scientific identity building highly divergent across student demographics (Belova et al., 2024). Design thinking approaches involving collaborative learning have emerged as effective methods in improving self-efficacy, as research studies have found that such approaches can mitigate demographic differences in science efficacy building (Santos et al., 2025). Research participation approaches lead to a marked improvement in efficacy compared to learning in science labs, thus underpinning the crucial need for participation in scientific work in building science efficacy in students (Newell and Ulrich, 2022).

Metacognitive strategies are pivotal regulatory activities through which students regulate, analyze, or control their cognitive actions in knowledge-building activities. Evidence has been found for bidirectional associations between learning with metacognitive strategies and self-efficacy beliefs, in which understanding of strategic knowledge is a joint cause of self-efficacy beliefs (Arianto and Hanif, 2024). This need for clear metacognitive preparation was brought to the forefront in a pandemic-induced transition to online learning environments, where students possessing metacognitive knowledge outperformed those lacking in terms of adaptability in new conditions (Anthonysamy, 2021). Pedagogical interventions for improving strategic understanding in life science learning have led to marked gains in terms of process and performance components (Stanton et al., 2021). Professional learning activities that have extended metacognitive thinking to science teachers’ learning have proved that teachers’ metacognitive learning directly correlates to their ability to develop similar skills in students (Chen et al., 2025).

Structural equation modeling studies have also shed light upon the complex interrelationships that exist between self-efficacy, metacognitive strategies, and learning achievements. Research studies have concluded that self-efficacy is a factor that impacts academic achievements in a multi-faceted manner, through both direct and indirect paths, influencing metacognitive strategy use and emotion regulation as a mediating variable (Hayat et al., 2020). The mediating effect of metacognitive strategies was also supported by a study that found concrete proof that improving self-efficacy expectations would lead to an improvement in metacognitive skills, which in turn facilitates greater learning engagement (Hayat and Shateri, 2019). A synthesis of theories casts self-regulation as a point of interaction between metacognitive control and self-process or motivation in a manner that treats them as interwoven variables in a non-independent relationship (Zimmerman and Moylan, 2009). This theoretically integrated position suggests that self-regulation is a process that develops as an interaction of beliefs in one’s capabilities related to learning.

Mixed learning contexts introduce specific affordances and difficulties in self-regulatory learning development. Replicated summaries of research studies have uncovered challenges in implementing blended learning such as demands in pedagogical redesign, technological infrastructure needs, and necessity in institutional support needs (Anthony et al., 2022). Studies that examine integrated versus distributed approaches in blended learning have uncovered implications of blended learning approaches in students’ perceptions of learning connectivity (Platonova et al., 2022). Recent studies have found that teachers’ data literacy is crucial in improving responsive teacher scaffolding in science blended learning approaches (Hanč et al., 2024). Research profiles have uncovered students’ heterogeneous self-regulatory behaviors in blended learning approaches in adults, signifying that students need blended learning strategies based on diverse self-regulatory learning approaches (Van Laer and Elen, 2020). Multivariates have supported that self-efficacy, self-regulated learning, and self-regulated learning components are interrelated predictions in academic achievements in studies (Zheng et al., 2021), while recent research studies have validated that technology proficiencies mediate interaction pattern relationships in self-regulated learning in digital learning systems (Hashmi et al., 2025).

## 3 Theoretical framework and hypotheses

### 3.1 Theoretical framework construction

This research develops an integrative theoretical framework based on self-regulated learning theory, which views academic accomplishment as a product of a cognitive, metacognitive, and motivational process interwoven through self-regulated learning activities. Zimmerman’s self-regulation cycle offers a basic architecture that envisions learning as taking place in a cycle of activities including planning, performance, and reflection upon those performances (Zimmerman and Moylan, 2009). Applications of this cycle in recent studies have supported its validity within varied learning scenarios (Zheng et al., 2021). In this self-regulation cycle, self-efficacy is seen as a powerful motivational antecedent that predicts learners’ setting of learning goals, strategy use, and persistence when tackling a learning task. Metacognitive strategies are seen as regulatory variables that use planning, monitoring of learning activities, and self-evaluation of activities toward learning objectives. This framework of self-regulated learning is further complicated by a blended learning environment, in which students have to regulate their learning activities through synchronous and asynchronous learning approaches that have unique learning challenges in a physical as well as an online classroom setting. In fact, this framework depicts self-efficacy as a dynamic learning element that is involved in a two-way interaction with metacognitive learning variables, in which efficacious learners demonstrate a greater propensity for deploying advanced metacognitive strategies, while successful strategy implementation reciprocally reinforces self-efficacy beliefs through accumulated mastery experiences. It is important to acknowledge that Zimmerman’s theoretical framework conceptualizes self-regulated learning as an inherently cyclical and temporally unfolding process encompassing forethought, performance, and self-reflection phases. The present study, employing a cross-sectional design, captures a single temporal snapshot of these interrelationships rather than directly modeling the recursive dynamics theorized by Zimmerman. Accordingly, the structural paths examined herein represent the concurrent directional associations among self-efficacy, metacognitive strategies, and achievement at a given point in the self-regulatory cycle, providing empirical support for the hypothesized variable relationships while recognizing that the full cyclical nature of these processes necessitates longitudinal verification in future research.

**Figure 1:**
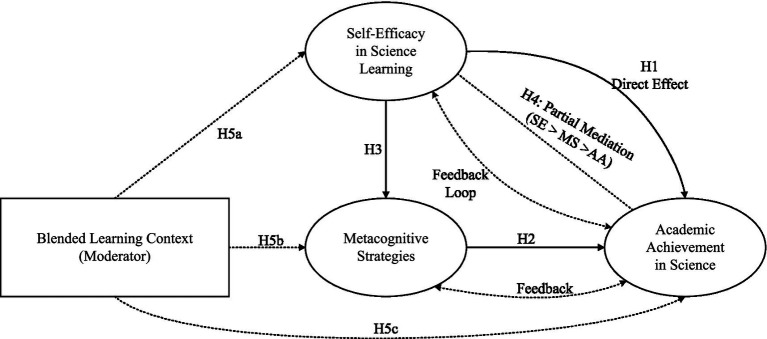
Conceptual model of self-efficacy, metacognitive strategies, and academic achievement in blended science learning.

The theoretical integration thus conceptualizes effective science learning in blended environments as contingent upon the synchronized development of motivational beliefs and strategic competencies (Figure 1).

### 3.2 Conceptual model of variable relationships

Figure 1 above shows that the conceptual framework outlines several paths through which self-efficacy and use of metacognitive strategies affect blended learning outcomes in science education. Self-efficacy has a direct effect on academic performance through its motivational and effort-allocation components throughout the learning process (Hayat et al., 2020; der Von Mehden et al., 2025). On a concurrent platform, self-efficacy also indirectly affects performance through its relationship with use of metacognitive strategies (Hayat and Shateri, 2019; Hayat et al., 2020), as students with higher self-efficacy tend to be more receptive to cognitive regulatory activities. Metacognitive strategies act as a mediator between self-efficacy and performance, as strategy use transforms motivational influence into action (Arianto and Hanif, 2024). This blended learning context serves as a moderator that moderates relationships defined in this framework (Van Laer and Elen, 2020). In online learning, autonomy is at its peak, and it is likely that the relationship between self-efficacy beliefs and use of metacognitive strategies is strengthened as students control their learning schedules. Face-to-face interactions, in their social aspect, may act as a scaffold supporting performance while offsetting low self-efficacy perceptions. Both self-efficacy and metacognitive strategy use have a positive effect on performance through a positive performance outcome, thus enabling a positive cycle of self-regulation skills acquisition (Hashmi et al., 2025; Zheng et al., 2021).

### 3.3 Research hypotheses

Building upon the theoretical framework and conceptual model presented in Figure 1, this study advances the following hypotheses. H1: Self-efficacy in science learning directly predicts academic achievement in blended learning environments, with students reporting higher efficacy demonstrating superior performance outcomes independent of metacognitive influences. H2: Metacognitive strategy use positively predicts academic achievement, as learners who systematically plan, monitor, and evaluate their learning activities attain higher levels of mastery. H3: Self-efficacy positively influences metacognitive strategy deployment, with efficacious learners exhibiting more frequent and sophisticated use of regulatory strategies. H4: Metacognitive strategies partially mediate the relationship between self-efficacy and academic achievement, such that self-efficacy’s effect on performance operates both directly and indirectly through its influence on strategic behavior. The theoretical rationale for hypothesizing online learning engagement as a moderator resides in the distinct self-regulatory demands imposed by online learning modalities. In online learning environments, students operate with diminished external scaffolding and heightened autonomy requirements, creating conditions under which self-efficacy beliefs become particularly consequential for initiating and sustaining metacognitive regulatory activities (Van Laer and Elen, 2020). Specifically, highly engaged online learners encounter more frequent self-regulatory decision points, such as determining when to review material, how to allocate study time, and whether comprehension has been achieved, that require the autonomous activation of metacognitive strategies to a degree not typically demanded in instructor-directed face-to-face settings. The increased autonomy inherent in online learning thus amplifies the functional pathway from self-efficacy to metacognitive strategy deployment, as self-efficacious learners in high-autonomy online contexts are more likely to translate their confidence into active planning, monitoring, and regulation of learning activities, whereas learners with lower online engagement operate within more externally structured environments where metacognitive activation is partially scaffolded by instructors and peers, thereby attenuating the direct motivational impetus from self-efficacy to strategy use. H5: Online learning engagement moderates the relationship between self-efficacy and metacognitive strategy deployment, such that the positive influence of self-efficacy on metacognitive strategies is stronger among students with higher levels of online engagement, reflecting the heightened self-regulatory demands and autonomy requirements inherent in online learning contexts. Collectively, these five hypotheses position self-efficacy as both a direct determinant of academic success and an indirect influence operating through metacognitive pathways (H1–H4), while positing online learning engagement as a contextual boundary condition that selectively modulates the motivational-strategic linkage (H5).

## 4 Methodology

### 4.1 Research design

This research work utilized a quantitative correlational study to investigate linkages between self-efficacy, metacognitive strategies, and academic performance in blended learning science environments. This study endeavored to validate a proposed mediation model that hypothesized metacognitive strategies as a mediator of self-efficacy and academic performance, moderated by learning environment variables. This study aimed to validate a proposed model within a defined research framework that was validated through crosses-sectional surveys and institutional data in the spring of 2024. This research was designed to validate five study hypotheses (H1-H5) within a self-regulated learning theory framework, while utilizing Structural Equation Modeling techniques to analyze direct, indirect, and moderating paths within study data.

### 4.2 Participants and sampling

Participants included 463 college students enrolled in blended introductory science courses (biology, chemistry, physics) at two public universities located in Guangdong Province, China. Participants were conveniently sampled based on criteria that included at least one semester (16 weeks) of blended learning experience. The sample included 278 females (60.0%) and 185 males (40.0%), aged 18–22 years (*M* = 19.4, SD = 1.2). Participants were distributed across academic years: first-year (43.2%), second-year (35.6%), and third-year (21.2%). Informed consents were sought from all participants after clearance by an institutional ethics committee (Protocol #2024-EDU-018). A total of 449 students were considered in this study after excluding 14 students with incomplete responses, well above the number needed in a Structural Equation Modeling analysis. The use of convenience sampling from two universities within a single province is acknowledged as a constraint on the generalizability of findings. This sampling strategy was adopted to ensure comparability in blended learning infrastructure and curricular designs across participating institutions, thereby minimizing confounding institutional variability that could obscure the self-regulatory mechanisms under investigation. The demographic composition of the sample, encompassing students across three academic years and three science disciplines of biology, chemistry, and physics, provides reasonable within-sample heterogeneity. Nevertheless, caution is warranted in generalizing these findings beyond the specific institutional and cultural context of Guangdong Province, and cross-regional replication studies are needed to establish broader external validity. These implications are further discussed in the limitations section.

### 4.3 Blended science learning environment

This blended learning environment involved a combination of face-to-face learning and online learning activities organized according to a rotation cycle. There were two 90-min face-to-face learning sessions per week that focused on such activities as collaborative inquiry work, lab experiments, problem-solving discussions, and instructor-led feedback. Online learning activities involved three learning modules per week that utilized Blackboard’s learning system with such elements as video presentations of scientific concepts, simulations, online labs, self-evaluation tests, and online discussion activities. Students could generally take part in independent online learning activities according to individual schedules that average 4 to 5 h a week. The approximate proportion of instructional time allocated to face-to-face and online components was 40:60, with approximately 3 h per week devoted to in-person sessions (two 90-min meetings) and four to 5 h designated for asynchronous online learning activities. The online component was delivered primarily through the Blackboard learning management system, which hosted video lectures on scientific concepts, interactive simulations, virtual laboratory exercises, self-assessment quizzes, and asynchronous discussion forums. Face-to-face sessions were conducted in science laboratories and seminar rooms, focusing on collaborative inquiry, hands-on experimentation, and instructor-facilitated discussion. This blended configuration aligns with the rotation model described in contemporary blended learning taxonomies (Istenič, 2024), wherein students alternate systematically between online self-directed learning and in-person collaborative activities within a structured weekly cycle. This learning environment mimics real-word science learning instruction in such a way that students’ self-regulated learning activities in collaborative and independent learning domains could be inspected for self-efficacy and strategy usage.

### 4.4 Instruments and measures

#### 4.4.1 Self-efficacy scale

Science learning self-efficacy was assessed through an 8-item scale designed from the Motivated Strategies for Learning Questionnaire (MSLQ). This was a validated measure for evaluating self-efficacy in a particular field of study in a learning environment (Hayat et al., 2020). This was modified to fit science learning in a blended setting. Students were asked how confident they were in understanding scientific ideas, performing scientific experiments, answering scientific problems, and performing well in a blended classroom setup. Students responded based on a 7-point likert scale ranging from 1 (“not at all confident”) to 7 (“completely confident”). Examples of questionnaire sentences include “I am confident that I can readily understand complex scientific concepts covered in this course” and “I am capable of independently performing science assignments in online classrooms.” The scale demonstrated excellent internal consistency reliability (Cronbach’s *α* = 0.89, M = 5.21, SD = 1.06). Higher scores indicated stronger self-efficacy beliefs.

#### 4.4.2 Metacognitive strategies scale

Metacognitive strategies were tapped through a 12-item adaptation of the Metacognitive Awareness Inventory (MAI) because of its sound psychometric properties and known efficacy in capturing metacognitive control in learning situations. The battery of items tapped three theoretically supported components of metacognitive control: planning (4 items), monitoring (4 items), and regulation (4 items). Planning was assessed through items related to learning objective formation and preparation (for instance, “I set specific learning objectives before learning science materials”). Monitoring was tapped through items aligned with understanding assessment and tracking while engaging in learning activities (for instance, “I often check my understanding while learning science materials”). Responses utilized a 5-point frequency scale (1 = never, 5 = always). Reliability coefficients were: planning (*α* = 0.84), monitoring (*α* = 0.86), regulation (*α* = 0.82), and total scale (*α* = 0.91, *M* = 3.68, SD = 0.74). It is recognized that self-report measures of metacognitive strategies are susceptible to social desirability bias, wherein students may overestimate the frequency and quality of their regulatory behaviors. To mitigate this concern, the present study employed several procedural safeguards: survey administration was conducted anonymously and independently of course evaluation processes, and participants were explicitly assured that their responses would not affect academic standing, thereby reducing incentives for socially desirable responding. Nevertheless, the reliance on self-report measures remains a limitation of the present study. Future research would benefit from triangulating questionnaire data with objective behavioral indicators derived from learning management system log data, such as login frequency, resource access patterns, and time-on-task metrics, to provide a more ecologically valid assessment of metacognitive engagement in blended learning environments.

#### 4.4.3 Science learning achievement

Academic performance was measured by using final grades that summed up a comprehensive assessment of learning in science. Academic performance was calculated based on scores from standardized exams (60%), lab reports (20%), project assignments (15%), and class participation (5%). The exams tested understanding, skills in solving problems, and application of scientific principles using multiple-choice, short-answer, and essay formats. Lab reports tested skills in performing scientific experiments and scientific reasoning. Project assignments tested skills in integration of scientific knowledge. Each score was normalize (*M* = 0, SD = 1) to make it possible to use it in a study that involved multiple courses and teachers. Scores from academic performance were standardized and had a range of −2.84 to 2.67 (*M* = 0, SD = 1.00).

### 4.5 Data collection procedures

Data collection was done in the latter 3 weeks of a spring 2024 semester after students completed 14 weeks of blended learning. Online survey research was conducted using a learning management system at our university supported by Qualtrics survey software. Invitations containing study details and anonymous survey links were sent to participants through their university email accounts. Students voluntarily participated in research activities out of class hours, taking around 25–30 min to complete. To link survey data to institutional achievements while ensuring participant anonymity, anonymous identification codes were utilized in data collection. End of semester grades for students were collected from university registrars after completing a semester. Response rate was 78.4% (463 of 590 students were invited).

### 4.6 Data analysis methods

In data analysis, Structural Equation Modeling was done employing Mplus 8.3. In preliminary analysis, descriptive statistics, Correlation matrices, analysis of missing data, and diagnostic checks (Normalities, Linearity, Multicollinearity) were done. Confirmatory factor analysis was done to verify Measurement models of Self-efficacy and Metacognitive strategies before analyzing Structural relationships. In the structural model, we tested (1) direct effects of self-efficacy on achievement (H1) and metacognitive strategies (H3); (2) direct effects of metacognitive strategies on achievement (H2); and (3) mediation through indirect effects with bias-corrected bootstrapping with 5,000 resamples (H4). For testing the moderating effect of learning environment characteristics (H5), multi-group SEM was performed by splitting participants into two groups according to their patterns of engagement with online learning, students highly engaged in online learning (≥ 5 h per week in online modules, *n* = 233) and students less engaged in online learning (< 5 h per week in online modules, *n* = 216). The five-hour threshold for differentiating engagement groups was determined through a combination of pedagogical and empirical considerations. The instructional design of the blended courses designated approximately four to 5 h per week as the expected online learning workload. Research on blended learning design has established that replacing 50–60% of face-to-face instruction with online activities represents an evidence-based proportion for effective blended courses (Alammary, 2022; Müller and Mildenberger, 2021), and the five-hour criterion thus corresponds to the upper boundary of the pedagogically prescribed online engagement level within this design framework. Moreover, this threshold yielded approximately balanced group sizes (*n* = 233 versus *n* = 216), which optimizes statistical power for multi-group structural equation modeling comparisons. Methodological research has demonstrated that when a theoretically motivated split point coincides with the central tendency of the distribution, dichotomization retains analytical integrity for group-based comparisons (Iacobucci et al., 2015). Additionally, given that the sample-reported mean online engagement was approximately 4 to 5 h weekly, the five-hour threshold corresponds closely to the central tendency of the distribution, providing a theoretically meaningful and statistically efficient split point. Chi-square difference tests evaluated differences between constrained (path estimates were equal across groups) and unconstrained models in terms of moderating effects. Criteria for model fit were considered as CFI > 0.95, TLI > 0.95, RMSEA < 0.06, SRMR < 0.08. Robust standard error estimates using maximum likelihood dealt with non-normality issues.

### 4.7 Reliability and validity

Instrument reliability was confirmed through internal consistency analysis. Cronbach’s alpha coefficients exceeded the recommended 0.70 threshold for all scales: self-efficacy (*α* = 0.89), metacognitive strategies planning (*α* = 0.84), monitoring (*α* = 0.86), regulation (*α* = 0.82), and total metacognitive strategies (*α* = 0.91). Confirmatory factor analysis affirmed construct validity, with all factor loadings above 0.60 (range 0.68 to 0.85), denoting robust correlations between latent variables and their indicators. Results supported convergent validity, in that positive correlations emerged between theoretically aligned constructs (*r* = 0.42 to 0.67, *p* < 0.001). Discriminant validity was supported by chi-square difference tests of constrained versus unconstrained models, as it was determined that constructs diverged in a meaningful manner. Finally, common method variance was assessed by Harman’s single-factor test, such that no single factor explained most of the variance (greatest factor = 34.8%).

To establish the comparability of measurement models across high and low engagement groups prior to multi-group structural equation modeling, a sequential measurement invariance analysis was conducted following established protocols (Cheung and Rensvold, 2002; Leitgöb et al., 2023). Configural invariance was first examined by fitting the measurement model simultaneously across both groups without equality constraints, yielding acceptable fit (*χ*^2^ = 575.59, df = 328, CFI = 0.955, TLI = 0.948, RMSEA = 0.052, SRMR = 0.046). Metric invariance was subsequently assessed by constraining factor loadings to equality across groups; the resulting model showed minimal change in fit (*χ*^2^ = 597.17, df = 344, ΔCFI = 0.002, ΔRMSEA = 0.001), well within the recommended criterion of ΔCFI ≤ 0.010 for establishing metric invariance (Cheung and Rensvold, 2002). The chi-square difference test was not statistically significant (Δ*χ*^2^ = 21.58, Δdf = 16, *p* > 0.05). These results confirm that the latent constructs were measured equivalently across engagement groups, providing a valid foundation for interpreting cross-group differences in structural path coefficients as substantive moderating effects rather than measurement artifacts.

It should be noted that online learning engagement was assessed through students’ self-reported estimates of weekly hours spent on online learning modules. While this measure captures students’ perceived engagement levels, it is subject to recall and estimation biases inherent in retrospective self-report. Future studies should consider supplementing self-reported engagement data with objective learning analytics derived from learning management system logs, such as cumulative login duration, module completion rates, and interaction frequency, to provide more precise engagement operationalization.

## 5 Results

### 5.1 Descriptive statistics and correlations

Table 1 above displays descriptive statistics and correlations for all variables in this study. The distribution of all variables was found to be nearer normal with a skewness and kurtosis of ±2, thereby justifying the use of maximum likelihood estimation for all subsequent models. Mean self-efficacy scored 5.21 (SD = 1.06) out of 7, while metacognitive strategy scored 3.68 (SD = 0.74) out of 5, showing that participants scored above average in self-efficacy and strategic use of metacognitive activities.

**Table 1:** Descriptive statistics and correlations among study variables (*N* = 449).

Variable	*M*	SD	Skewness	Kurtosis	1	2	3	4	5	6
1. Self-efficacy	5.21	1.06	−0.47	0.38	—					
2. Planning	3.75	0.81	−0.32	−0.21	0.51**	—				
3. Monitoring	3.68	0.79	−0.28	0.15	0.49**	0.56**	—			
4. Regulation	3.61	0.82	−0.19	−0.08	0.41**	0.49*	0.61**	—		
5. Metacognitive strategies (total)	3.68	0.74	−0.27	0.11	0.52**	0.82**	0.84**	0.81**	—	
6. Academic achievement	0.00	1.00	−0.23	0.31	0.43**	0.38**	0.35**	0.29*	0.39**	—

M, Mean; SD, Standard Deviation. Metacognitive strategies (total) represents the composite score of planning, monitoring, and regulation subscales. Academic achievement scores were standardized (*M* = 0, SD = 1). *p < 0.05; **p < 0.01.

Correlation analysis showed theoretically valid patterns. Self-efficacy was positively related to metacognitive strategy use (*r* = 0.52, *p* < 0.01) and academic achievement (*r* = 0.43, *p* < 0.01), offering initial support for hypothesis 1 and hypothesis 3. Metacognitive strategy use was positively related to academic achievement (*r* = 0.39, *p* < 0.01), offering preliminary support for hypothesis 2. Among metacognitive strategy components, intercorrelations were significant and substantial between monitoring and regulation (*r* = 0.61, *p* < 0.01), as well as between planning and monitoring (*r* = 0.56, *p* < 0.01) and planning and regulation (*r* = 0.49, *p* < 0.05). This supported their identification as distinct components of metacognitive self-regulation.

### 5.2 Measurement model validation

Confirmatory factor analysis was conducted to validate the measurement models before examining structural relationships. As shown in Table 2, the self-efficacy scale demonstrated excellent fit as a single-factor model (*χ*^2^ = 38.72, df = 20, CFI = 0.972, TLI = 0.964, RMSEA = 0.046, SRMR = 0.031). All factor loadings were greater than 0.70, ranging from 0.72 to 0.85, thus ensuring a good convergence of items to the construct self-efficacy. Factors of metacognitive strategies fit well in a three-factor model for planning, control, and regulating (*χ*^2^ = 89.54, df = 51, CFI = 0.968, TLI = 0.959, RMSEA = 0.041, SRMR = 0.038) with factor loadings ranging from 0.68 to 0.83. The overall measurement model, incorporating all latent constructs, also achieved satisfactory fit (*χ*^2^ = 328.91, df = 164, CFI = 0.961, TLI = 0.954, RMSEA = 0.047, SRMR = 0.042). These results confirmed that the measurement instruments possessed adequate psychometric properties, establishing a valid foundation for subsequent structural equation modeling analyses.

**Table 2:** Fit indices for measurement models (*N* = 449).

Model	*χ* ^2^	df	*χ*^2^/df	CFI	TLI	RMSEA	RMSEA 90% CI	SRMR
Self-efficacy (single-factor)	38.72	20	1.94	0.972	0.964	0.046	[0.023, 0.067]	0.031
Metacognitive strategies (three-factor)	89.54	51	1.75	0.968	0.959	0.041	[0.027, 0.055]	0.038
Overall measurement model	328.91	164	2.01	0.961	0.954	0.047	[0.040, 0.055]	0.042

*χ*^2^, chi-square; df, degrees of freedom; CFI, comparative fit index; TLI, Tucker-Lewis index; RMSEA, root mean square error of approximation; CI, confidence interval; SRMR, standardized root mean square residual. All factor loadings ranged from 0.68 to 0.85, exceeding the recommended threshold of 0.60. Recommended cutoff criteria: CFI/TLI ≥ 0.95, RMSEA ≤ 0.06, SRMR ≤ 0.08.

### 5.3 Structural model testing and hypothesis verification

**Figure 2:**
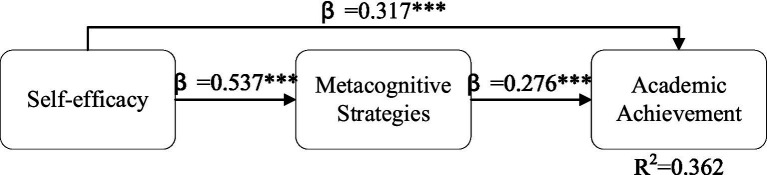
Structural model with standardized path coefficients. Standardized path coefficients shown. ***p < 0.001. *N* = 449. Model fit: CFI = 0.959, TLI = 0.952, RMSEA = 0.048, SRMR = 0.044.

Structural equation modeling was carried out to test the proposed relationships between self-efficacy, metacognitive strategies, and academic achievements. The structural model showed a good fit to the data (*χ*^2^ = 341.26, df = 167, CFI = 0.959, TLI = 0.952, RMSEA = 0.048, SRMR = 0.044), which showed that the proposed theoretical model was a good fit to the data. As shown in Table 3 and Figure 2, all three paths in the proposed model were statistically significant. Self-efficacy was a significant positive predictor of academic achievements (*β* = 0.317, SE = 0.049, *p* < 0.001) and supported hypothesis H1. Consistent with H2, metacognitive strategies significantly predicted academic achievement (*β* = 0.276, SE = 0.052, *p* < 0.001). Self-efficacy also demonstrated a strong positive influence on metacognitive strategies (*β* = 0.537, SE = 0.042, *p* < 0.001), confirming H3. Collectively, self-efficacy and metacognitive strategies explained 36.2% of the variance in academic achievement (*R*^2^ = 0.362).

**Table 3:** Structural model path coefficients and hypothesis testing results (n = 449).

Hypothesis	Path	*β*	SE	*t*-value	*p*-value	95% CI	Result
H1	Self-efficacy → academic achievement	0.317	0.049	6.47	< 0.001	[0.221, 0.413]	Supported
H2	Metacognitive strategies → academic achievement	0.276	0.052	5.31	< 0.001	[0.174, 0.378]	Supported
H3	Self-efficacy → metacognitive strategies	0.537	0.042	12.79	< 0.001	[0.455, 0.619]	Supported

*β*, standardized path coefficient; SE, standard error; CI, confidence interval. Model fit indices: *χ*^2^ = 341.26, df = 167, CFI = 0.959, TLI = 0.952, RMSEA = 0.048 [0.041, 0.055], SRMR = 0.044. The model explained 36.2% of variance in academic achievement (*R*^2^ = 0.362).

### 5.4 Mediation analysis

Mediation analysis using bias-corrected bootstrapping (5,000 resamples) examined whether metacognitive strategies mediated the relationship between self-efficacy and academic achievement (H4). As shown in Table 4, the total effect of self-efficacy on achievement was significant (*β* = 0.439, *p* < 0.001). Including metacognitive strategies as a mediator, the direct relationship of self-efficacy to academic success was also significant (*β* = 0.317, *p* < 0.001) while the indirect effect was also significant [*β* = 0.122, 95% CI (0.071, 0.181), *p* < 0.01]. In this case, it is clear that the 95% CI of the indirect effect was not zero, thus supporting significant mediation. This indicates that hypothesis 4 is supported and that metacognitive strategies partially mediate self-efficacy in regards to academic success by explaining 27.8% of the total effect.

**Table 4:** Mediation analysis: decomposition of effects (*n* = 449).

Effect type	Path	*β*	SE	95% CI	Significance
Total effect	Self-efficacy → Academic achievement	0.439	0.045	[0.351, 0.527]	*p* < 0.001
Direct effect	Self-efficacy → Academic achievement	0.317	0.049	[0.221, 0.413]	*p* < 0.001
Indirect effect	Self-efficacy → Metacognitive strategies → Academic achievement	0.122	0.028	[0.071, 0.181]	*p* < 0.01
Component path a	Self-efficacy → Metacognitive strategies	0.537	0.042	[0.455, 0.619]	*p* < 0.001
Component path b	Metacognitive strategies → Academic achievement	0.276	0.052	[0.174, 0.378]	*p* < 0.001

*β*, standardized coefficient; SE, standard error based on 5,000 bootstrap samples; CI, bias-corrected bootstrap confidence interval. The indirect effect is significant as the 95% CI does not include zero. Proportion of mediation: 27.8% (indirect effect / total effect = 0.122/0.439).

### 5.5 Moderation analysis

Multi-group SEM was carried out to find that online learning engagement was a moderator in relationships of study variables (H5). Participants were classified as high engagement group (n = 233, ≥ 5 h per week) and low engagement group (*n* = 216, < 5 h per week). As shown in Table 5, the path from self-efficacy to metacognitive strategies was significantly stronger in the high-engagement group (*β* = 0.592, *p* < 0.001) compared to the low-engagement group (*β* = 0.469, *p* < 0.001), with chi-square difference tests confirming significant moderation (Δ*χ*^2^ = 5.38, df = 1, *p* = 0.020). However, paths from self-efficacy to achievement and from metacognitive strategy use to achievement were not significantly different across groups (*p* > 0.05). The findings offer a partly supportive result for hypothesis H5 that online learning engagement as a moderating variable mainly impacts the relationship between self-efficacy and use of metacognitive strategies. A noteworthy finding from the multi-group analysis is that the self-efficacy-metacognitive strategies pathway remained statistically significant in the low-engagement group (*β* = 0.469, p < 0.001), albeit at a reduced magnitude compared to the high-engagement group (*β* = 0.592).

**Table 5:** Multi-group analysis: path coefficients by online learning engagement level (*n* = 449).

Path	High engagement (*n* = 233)	Low engagement (*n* = 216)	Δ*χ*^2^	Δdf	*p*-value	Moderation
H1: Self-efficacy → Academic achievement	0.334*** (0.052)	0.298*** (0.069)	1.47	1	0.225	Not significant
H2: Metacognitive strategies → Academic achievement	0.289*** (0.057)	0.253** (0.076)	1.21	1	0.271	Not significant
H3: Self-efficacy → Metacognitive strategies	0.592*** (0.046)	0.469*** (0.062)	5.38	1	0.020	Significant

Values are standardized path coefficients with standard errors in parentheses. High engagement = students spending ≥5 h weekly on online modules; Low engagement = students spending <5 h weekly. Δ*χ*^2^ = chi-square difference between constrained and unconstrained models; Δdf, degrees of freedom. Model fit for high engagement group: CFI = 0.961, TLI = 0.954, RMSEA = 0.047, SRMR = 0.042. Model fit for low engagement group: CFI = 0.957, TLI = 0.949, RMSEA = 0.052, SRMR = 0.047. **p < 0.01; ***p < 0.001.

## 6 Discussion

This study investigated interrelationships between self-efficacy, metacognitive strategies, and academic achievement in blended science learning environments. Results showed that self-efficacy not only predicts academic achievement directly but also predicts it indirectly through metacognitive strategies, with online learning engagement as a moderating variable.

The direct relationship between self-efficacy and academic achievements (*β* = 0.317) is supported by recent findings in the field of study (Basileo et al., 2024; Dunbar-Wallis et al., 2024). Also, the positive influence of metacognitive strategies on academic achievements (*β* = 0.276) supports meta-analytic findings for online blended learning (Zhao et al., 2025), showing that metacognitive strategies have a crucial influence in blended learning. It is noteworthy, however, that the magnitude of the self-efficacy-achievement path coefficient (*β* = 0.317) in the present study is moderate rather than large. This may reflect the distinctive characteristics of the blended modality, wherein face-to-face instructional components, including laboratory-based mastery experiences, collaborative inquiry activities, and direct instructor feedback, provide compensatory achievement mechanisms that partially buffer the consequences of lower self-efficacy. In exclusively online environments, where such compensatory scaffolds are absent, the direct effect of self-efficacy on achievement would be expected to be more pronounced. This interpretation suggests that the blended format may function as a protective instructional context that attenuates achievement disparities attributable to motivational differences, an observation that carries implications for equitable instructional design.

On performing mediation analysis, it was revealed that metacognitive strategies partially mediate the relationship of self-efficacy with achievement, explaining 27.8% of the total effect. This appears to corroborate findings related to self-regulation as proposed by Halmo et al. (2024), as well as findings related to longitudinal support for mediation as proposed by Kleka et al. (2024). This pattern of partial mediation suggests that self-efficacy has a twofold effect, either directly or indirectly through metacognitive strategies.

Online engagement significantly moderated the self-efficacy-metacognitive strategies pathway (Δ*χ*^2^ = 5.38, *p* = 0.020), with stronger associations observed among high-engagement students (*β* = 0.592) compared to low-engagement students (*β* = 0.469). This is supported by systematic review evidence of context effects in online learning (Broadbent and Poon, 2015), as well as Raharjo et al.’s (2025) findings regarding the effect of classroom environment variables on metacognitive strategies. This pattern indicates that self-efficacy retains its motivational function in activating metacognitive processes irrespective of engagement level, yet the attenuated effect size in the low-engagement group warrants interpretation. Students with lower online engagement operate predominantly within the externally scaffolded face-to-face instructional context, where instructor guidance and structured collaborative activities may partially substitute for the autonomous metacognitive regulation that online environments necessitate. In such instructor-directed settings, the translation of self-efficacy beliefs into self-initiated metacognitive behaviors is less imperative because external scaffolding compensates for individual differences in regulatory capacity. Regarding the allocation of time not invested in online modules, low-engagement students may substitute alternative learning activities, such as textbook-based review, peer discussion outside digital platforms, or passive content consumption, that impose fewer autonomous metacognitive demands compared to the self-paced, self-monitored online learning activities. This differential pattern supports the interpretation that the online learning context functions as a catalytic environment that amplifies the translation of motivational beliefs into active metacognitive deployment, consistent with the theoretical rationale underlying H5. An absence of moderation in the remaining paths suggests that online engagement uniquely strengthens the self-efficacy-strategies link. The non-significant moderation of the metacognitive strategies-to-achievement and self-efficacy-to-achievement pathways constitutes a theoretically informative finding that warrants careful interpretation rather than dismissal. One plausible explanation is that the direct effects of both self-efficacy and metacognitive strategies on achievement operate through mechanisms that are relatively invariant across engagement levels, as the motivational persistence conferred by self-efficacy and the executive regulation enabled by metacognitive strategies may function comparably whether enacted in online or face-to-face contexts, because achievement outcomes ultimately depend on cumulative knowledge acquisition processes that are not modality-specific. In contrast, the activation of metacognitive strategies from self-efficacy beliefs, a process of translating motivation into regulatory action, is uniquely sensitive to the autonomy demands of the learning context. This selective moderation pattern implies that interventions targeting online engagement are most consequential at the motivational-strategic interface, where self-efficacy beliefs are converted into regulatory behavior, rather than at the strategy-performance junction where regulatory behaviors are translated into achievement outcomes.

The theoretical contributions of these findings can be summarized in three aspects. The partial mediation model demonstrates that self-efficacy operates through dual pathways in blended science learning, namely a direct motivational route that sustains effort and persistence, and an indirect strategic route mediated by metacognitive regulation. This dual-pathway architecture extends self-regulated learning theory by empirically differentiating the motivational and strategic functions of self-efficacy within a discipline-specific blended learning context. Additionally, the identification of online engagement as a selective moderator, specifically strengthening the self-efficacy-to-metacognition linkage while leaving the remaining pathways unaffected, refines current understanding of boundary conditions in self-regulated learning, indicating that contextual factors operate differentially across distinct self-regulatory sub-processes rather than uniformly across the entire model. Furthermore, while the cross-sectional design precludes direct verification of Zimmerman’s cyclical self-regulation process, the present findings provide empirical support for the concurrent directional associations predicted by the framework, establishing a foundation for longitudinal investigations that could more fully capture the temporal dynamics of self-regulatory cycles in blended science learning.

Results have several implications for science learning in a blended environment. First, teachers need to understand that enhancing self-efficacy beliefs may not be adequate because of its pivotal role in metacognitive strategy learning. Second, as supported by moderation results, learning designs need to take students’ engagement in online learning sessions seriously because self-efficacy beliefs and learning of metacognitive strategies in blended science learning are nonlinearly related.

There are a few limitations that need to be considered. First, because of its cross-sectional nature, it is not possible to make inferences of causation. Longitudinal studies need to be undertaken to establish precedence in terms of how a relationship develops. Second, because it is based upon self-reporting, it is vulnerable to common method variance. Future studies need to use behavioral traces to establish learning behaviors. Specifically, both metacognitive strategy use and online learning engagement were measured through self-report instruments, which may be subject to social desirability and recall biases. Incorporating objective indicators from learning management system logs, such as login frequency, time-on-task, and module completion sequences, would strengthen the validity of engagement and strategy use measurements in future research. Highly complex phenomenon, and it is recommended that engagement quality, in terms of interaction rate, use of resources, and cognitive presence in online learning, be considered in research studies in the future. In including only one type of student, that of Chinese undergraduates, it is clear that findings will not generalize across cultures and learning institutions. Cross-regional and cross-cultural replication studies involving diverse institutional contexts and student populations are essential to establish the external validity of the proposed dual-pathway model. It is also likely that unmeasured variables will act as extraneous variables in research studies.

Future studies may include metacognitive strategy interventions in blended learning approaches as proposed by Dignath and Büttner (2008). Experimental study designs with self-efficacy and metacognitive instruction may be conducted to identify causal relationships. Other studies may also investigate the progression of such underlying mechanisms at different stages of development and disciplines aside from science.

## 7 Conclusion

This study analyzed the interactions between self-efficacy and metacognitive strategies, as well as their influence upon blended science learning in a group of 449 Chinese undergraduates. In conclusion, the research showed that self-efficacy has a positive direct effect upon blended science learning (*β* = 0.317, *p* < 0.001) and an indirect effect through metacognitive strategies (*β* = 0.122, *p* < 0.01) of 27.8%. Multi-group analysis showed that online engagement has a significant moderating effect upon the relationship between self-efficacy and metacognitive strategies (Δ*χ*^2^ = 5.38, *p* = 0.020) in that high-engagement users have a greater relationship of 0.592 compared to low-engagement users at 0.469.

This research contributes to self-regulated learning theory, as it reveals how self-efficacy beliefs can be transformed in technology-enhanced learning environments. Finally, it appears that online engagement could be a boundary condition, as it is a factor that needs to be considered in learning activities in order to build self-efficacy and metacognitive skills in students. Future studies should use a longitudinal or intervention research approach in order to establish a cause-effect relationship. For science educators, these findings underscore the importance of adopting integrated instructional designs that simultaneously cultivate self-efficacy beliefs and metacognitive competencies, with particular attention to structuring online learning experiences that provide sufficient autonomy and engagement opportunities to activate the motivational-strategic pathway identified in this study.

## Data Availability

The raw data supporting the conclusions of this article will be made available by the authors, without undue reservation.
